# Evaluation by MALDI-TOF MS and PCA of the diversity of biosurfactants and their producing bacteria, as adaption to weathered oil components

**DOI:** 10.1016/j.btre.2021.e00660

**Published:** 2021-07-28

**Authors:** Shaikha Y. Alsayegh, Zulfa Al Disi, Mohammad A. Al-Ghouti, Nabil Zouari

**Affiliations:** Department of Biological and Environmental Sciences, College of Arts and Sciences, Qatar University, PO. Box 2713, Doha, Qatar

**Keywords:** Biosurfactants, Hydrocarbon-degrading bacteria, Weathered oil, MALDI-TOF MS, PCA

## Abstract

Indigenous Qatari bacterial strains were isolated from highly weathered oil-contaminated sites, identified, and differentiated based on their protein profiles using matrix-assisted laser desorption ionization-time of flight mass spectrometry (MALDI-TOF MS). Their diversity was demonstrated by the principal component analysis (PCA) analysis and establishment of a proteodendogram. Both were based on the protein profile of each strain. Interestingly, this approach also showed diversity within the same subspecies. This high diversity is reflected in the emulsification and solubilization activities of their extracellular biosurfactants. The highest emulsification activity (42.1 ± 2.11 AU/mL) was obtained with a strain of *Lysinibacillus fusiformis* (SA4) after one week of growth in the minimum salt medium in which diesel (5%) is the sole carbon source, while the highest solubilization activity (9.47% ± 0.47%) was produced by the strain *Bacillus subtilis* (SA6). The functional diversity of the biosurfactants was demonstrated by PCA analysis which allowed their further clustering based on the Fourier-transform infrared spectroscopy (FTIR) analysis. These findings clearly showed that two types of adaptations occur with hydrocarbons degrading bacteria in the weathered-oily soils, one related to the bacterial cell composition maintaining the biosurfactants composition and one to the biosurfactants, which are the primary tool employed by the cell to interact with the weathered oil. This finding would shed light on the potential and strategies of applications for the bioremediation of highly weathered oil-contaminated soils.

## Highlights

• MALDI-TOF MS MS and PCA allowed the identification and categorization of newly isolated strains from highly weathered oily soils.
• The isolated strains exhibited high diversity in terms of emulsification and solubilization activities.
• FTIR analysis combined with PCA revealed further diversity level of the produced biosurfactants.

## 1 Introduction

Microbial biosurfactants are surface-active molecules, which are produced by microorganisms, especially bacteria, to increase the bioavailability of hydrophobic substrates [1]. If appropriately produced and formulated, they can be applied in different fields ranging from hydrocarbons remediation to the food industry [2]. The surface-active biomolecules have unique properties such as low toxicity and a relatively simple preparation process [3]. Therefore, these characteristics have increased their demand in a wide range of industries of agrochemicals, fertilizers, petrochemicals, pharmaceuticals, cosmetics, and beverages as well as in petroleum and mining industries [4]. Biosurfactants have gained huge interest from the oil industry due to their property of reducing surface tension, which enables their use in bioremediation of oil pollution and oil recovery. In bioremediation, the major roles performed by biosurfactants include the increase of solubilization and desorption of the hydrophobic pollutants, increasing thus their bioavailability [5]. The future of bioremediation of soils contaminated by hydrocarbons is expected to be based on the surfactant-enhanced bioavailability of the contaminants. Biosurfactants have hydrophobic and hydrophilic sides, which lead to the formation of an interface region between fluids with different polarities such as water and oil hydrocarbons [6]. In comparison with other synthetic or chemical surfactants, biosurfactants have various advantages including their biodegradability, digestibility, and biocompatibility making them more effective in cleaning the environment and in the bioremediation of contaminated soils [7].

Biosurfactants are categorized based on their composition and origin. The molecules are grouped based on their molecular weight, namely high molecular weight (HMW) and low molecular weight (LMW). The low molecular weight biosurfactants include glycolipids [Trehalose lipids, rhamnolipids, Mannosylerythritol (MEL), Sophorose lipids, Mannosylarabitol lipid (MAL), Mannosylribitol lipid (MRL)], lipopeptides [Polymixins, surfactin, iturin lichenysin, fengycin, and serrwettin], and phospholipids [spiculisporic acid]. The high molecular weight biosurfactants include polymeric biosurfactants [Emulsans, Biodispesans, alasans, liposans] and particulate biosurfactants [vesicles, Sulphated polysaccharides, whole-cell, and food emulsifiers] [1]. Glycolipid biosurfactants are characterized by the property of a polysaccharide on their head groups. When the head group is affected by changes in pH or by electrolytes, the micellar structure of these biosurfactants changes [8]. Among the glycolipid biosurfactants, various hydrophobic fatty acids have the ability to reduce Kraft temperature at which the solubility of a surfactant matches the surfactant's critical micelle concentration (CMC) [1]. Then, fatty acids ensure that their structure is maintained. On the other hand, rhamnolipids are composed of rhamnoses and hydroxyl fatty acids [9]. Sophorolipids are made of sophorose, which forms the hydrophilic part of the surfactant [10]. The hydrophobic part is made of fatty acids that contain a long chain of carbon atoms [11]. Surfactin, a lipopeptide biosurfactant is composed of surfactin molecules that consist of seven amino acids and fatty acids that form the hydrophobic part. High molecular weight biosurfactants majorly function in the emulsification of hydrophobic molecules and they have a wide diversity in their structures and functions [8]. Biosurfactants are generated from microorganisms like *Bacillus, Pseudomonas,* and *Acenetobacter* genera among others. This is achieved through the process of fermentation and the enzyme-substrate reaction. Synthesis of biosurfactants is not only achieved intracellularly but can also be carried out extracellularly by the use of biocatalysts, which are enzymes. The hydrophilic and hydrophobic moieties can either be synthesized independently following different pathways and they can both be dependent on a substrate or one can be induced by the substrate and the others are synthesized a new [12]. In the amphiphilic structure of the biosurfactants, the hydrophobic moiety can be either a hydroxyl-fatty acid or a long-chained fatty acid. The hydrophilic moiety can be either a carboxylic acid, amino acid, carbohydrate, phosphate, alcohol, or cyclic peptide. The synthesis of these moieties involves the carbohydrate metabolic pathway, which synthesizes the hydrophilic moiety, and the hydrocarbon metabolic pathway, which synthesizes the hydrophobic moiety. In most cases, the first enzymes used in the process of precursor synthesis are regulatory enzymes [13]. Various factors influence biosurfactants synthesis and affect the rate of production and their properties. Some of the factors that influence the optimum production of biosurfactants include the carbon and nitrogen sources, temperature, pH, oxygen availability, carbon-nitrogen ratio, and agitation [14]. Consequently, all the reported parameters affect production, composition, and activity. However, in bioremediation approaches, the recalcitrance of certain petroleum compounds to biodegradation results from their strong adsorption on soil particles. Here, adsorption is understood as the retention of a solute in solution by the surface of a solid material, whereas adsorption refers to the retention of the solute within the mass of the solid [15]. The strength of this adsorption depends on the contaminant and the matrix on which it is adsorbed [16]. It is important to note that the climate of the gulf region is characterized by harsh conditions, resulting in accelerated weathering of oil components. Under such conditions, it is anticipated that indigenous microorganisms have adapted to synthesize specific biosurfactants that are effective for these weathered oils, both to mobilize the hydrocarbons and to enhance their bioavailability and biodegradation. Indeed, many failures of bioremediation applications in regions characterized by harsh weather and soils can be attributed to the use of un-acclimated bacteria and their associated biosurfactants [17]. The novelty of this work resides in the ability of the indigenous Qatari strains to produce biosurfactants that have the potential to enhance the biodegradation of weathered hydrocarbons or washing of weathered soil. These biosurfactants may be more active under the harsh physical and chemical conditions with Qatari soils, making them appropriate for use in enhanced oil recovery and bioremediation. However, it is necessary to demonstrate the biodiversity of the producing bacteria and strains, and their adaptation to harsh conditions and weathered hydrocarbons by adapting their biosurfactants structures and activities. A collection of bacteria was formed, and most of the isolates have been identified and differentiated, and their potential to produce biosurfactants has been evaluated. The isolates from the highly weathered oily sites would lead to selecting interesting strains more appropriate in applications in areas characterized by harsh weather. Their adapted biosurfactants were investigated by Fourier-transform infrared spectroscopy (FTIR) and categorized by principal component analysis (PCA). Indigenous bacteria would be highly adapted and when re-introduced or stimulated would conduct to the remediation of these sites.

## 2 Material and methods

### 2.1 Soil samples collection

Soil samples were collected from different areas in Qatar, characterized by aged pollution with petroleum hydrocarbons. Two dumpsites in Dukhan industrial area were selected because solid and liquid wastes from the oil industry were discharged and left for self-purification for more than three years in the open air. One sampling site was chosen away from the sea line and another in the intertidal zone of Dukhan. All these selected sites are characterized by pollution with weathered oil as previously demonstrated [18]. An automotive workshop in Doha industrial area was also selected with recent and aged pollution with diesel and lubricants. All samples were collected from the surface soil layer of 10 cm, homogenized in sterile 50 mL tubes and preserved at 4 ∘C until use (Table 1). The pH of the samples was ranging from 6.85 ± 0.05 to 7.15 ± 0.06. The moisture was of 8.6 ± 0.3 to 13.6 ± 0.5. Based on results of our previous work, the total petroleum hydrocarbons (TPH) and the oil range organics (ORO) contents of the samples were ranging were from 190 mg/kg to 325 mg/kg, and the TPH diesel range organic (DRO) contents were always less than 1 mg/kg g/kg (Alkaabi et al., 2020). Nitrate, ammonia and phosphate contents were below 0.140 mg/g, 0.007 mg/g and 0.350 mg/g respectively.

**Table 1:** Isolated hydrocarbon-degrading bacterial strains from the oily-soils sampled from different locations in Qatar.

Soil sample locations	Strains code
Dukhan, away from sea line (Dukhan Sealine)	SA2, SA3, SA4, S5
Dukhan, intertidal zone (Dukhan IZ*)*	SA9, SA10, SA11, SA12, SA14, SA16
Dukhan, site 1 of dumpsite (DS1)	SA6, SA17
Dukhan, site 2 of dumpsite (DS2)	SA28, SA29, SA31, S32, S33
Automotive Workshop (AWS)	S27, S24

### 2.2 Enrichment cultures with native bacteria

**Fig. 1:**
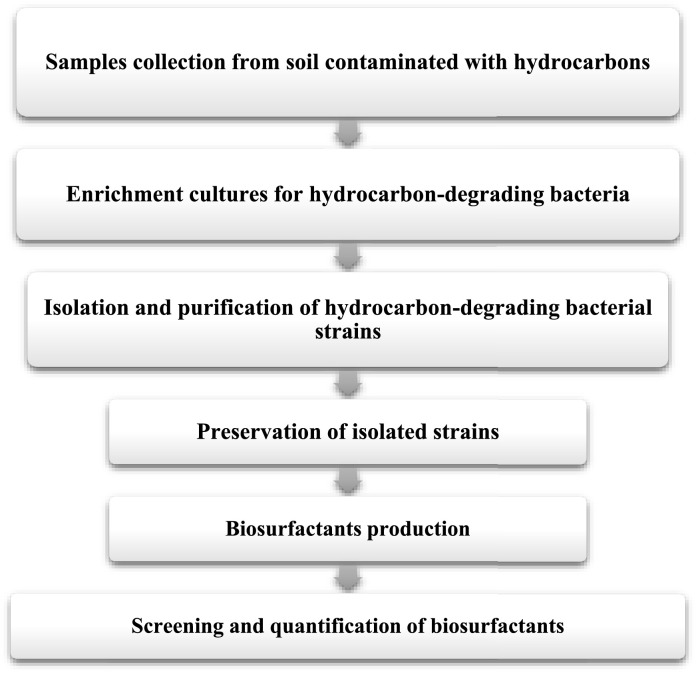
Strategy employed to isolate and screen biosurfactants producing bacteria.

Hydrocarbon-degrading bacteria were enriched from soil samples using a standard enrichment method [18, 19, 20] as shown in Fig. 1. In 50 mL sterile tube, 1 g of soil sample was added to 25 mL Minimal Salt Medium (MSM) that contains (w/v): 0.1% NH_4_Cl, 0.1% KH_2_PO_4,_ 0.4% Na_2_HPO_4_.2H_2_O, 0.006% KCl and 0.04% MgSO_4_.7H_2_O with pH set at 7.0 before sterilization. Before inoculation, the medium was supplemented with 1% (v/v) of a trace element solution. The trace element solution was composed of (g/100 mL): EDTA, 0.1; ZnSO_4_, 0.042; MnSO_4_, 0.178; H_3_BO_3_, 0.05; NiCl_2_, 0.1. All media were autoclaved for 20 min at 121°C. Solid MSM was obtained by adding 15 g/L agar. 5% (v/v) diesel – the sole carbon source- was then added in a final culture volume of 20 mL. Diesel stock was kindly provided by Mesaieed Refinery (Qatar) with a complete analysis, indicating hydrocarbons composition ranging from n-C_12_ to n-C_25_. It contained 750 g/L carbon. All cultures were incubated at 30°C for two weeks in a shaker set at 200 rpm. After one week of incubation, 2 mL of each culture was used to inoculate a fresh medium, as performed in the first culture. Three subsequent sub-culturing were then performed, before proceeding to the isolation of the enriched hydrocarbon-degrading bacteria.

### 2.3 Isolation and purification of bacterial strains from the enrichment cultures

100 µL of the enriched cultures were plated on MSM solid media and then coated with 100 µL diesel, spread on the surface of the plate which was then sealed with PARAFILM® and incubated at 30°C. Colonies of different aspects (form, color, shining, and size) were spread on LB solid agar plates. The isolated colonies were separately displaced by streaking in LB solid agar plates. Purification of the isolated strains was performed by six successive subculturing steps using separated colonies at each step. Strains were preserved at -80°C in LB medium containing 30% glycerol.

### 2.4 Sample processing and protein extraction for MALDI-TOF MS analysis

In order to produce the most reliable results, two techniques were used to prepare the samples for matrix-assisted laser desorption ionization-time of flight mass spectrometry (MALDI-TOF MS) [21, 22, 16] . The extraction was performed using equivalent volumes of ethanol and formic acid [23](Wang et al., 2012). A separate colony of a strain from an LB plate was suspended in 300 µL sterile water and then re-suspended in 900 µL absolute ethanol. After centrifugation at 10,000 rpm for 5 min, the pellet was supplemented with 1 mL of formic acid (70%) and then 1 mL acetonitrile (100%). After centrifugation, 1 µL of the supernatant was introduced into the biotarget of 48 sample spots. Then, 1 µL of alfa-cyano-4-hydroxycinnamic acid (HCCA) matrix solution containing (50% acetonitrile and 2.5% trifluoroacetic acid in ultra-pure water) was added for protein extraction. Triplicates were performed by spotting separate colonies in three wells. In parallel, a second method was performed using the whole bacterial cells. Here, one fresh and separate cell colony was transferred using a sterile loop into the well of the target plate. Subsequently, mass fingerprints were directly generated by the apparatus, allowing obtention of a MALDI-TOF MS score and a protein profile for each strain.

### 2.5 Identification of the bacterial isolates

The MALDI-TOF MS analysis was performed using a Brucker apparatus (Model: microflex LT/SH from Bruker Daltonics, Germany). The identification of the bacterial isolate was performed by the Bruker Biotyper software.

The mass spectra generated by MALDI-TOF MS were analyzed for similarities to the database entries.

Proteins with an *m/z* between 2,000 *m/z* and 20,000 *m/z z* are used to identify bacterial strains based on individual mass peaks corresponding to specific ribosomal proteins of distinct types of microorganisms, compared to a database available in the software. The mass peaks corresponding to specific ribosomal proteins are used for the identification of the isolates by similarities establishment. The results were in form of log(scores) as generated by default by the Biotyper software. A log scale from 0.000 to 3.000 was obtained for each strain. If the score ranges between 2.300 and 3.000, the identification is at the highly probable species level with high confidence. The scores ranging between 2.000 and 2.299 provide high accurate identification at the genus and probable correct at species level. The scores in the range between 1.700 -1.999 provide probable genus-level identification. The scoring system was provided by Bruker Daltonics / Germany, using the software MBT Compass version 4.1.80.24 (Data base version: MBT Compass Library Build 9.0.0.0, Revision: F). It is the most up to date scoring system as per the manufacturer guideline.

### 2.6 Data processing

The protein profiles were generated by the Bruker Flex Control software as mass spectra (linear and positive mode, at 60 Hz laser frequency and 35% intensity) using an acceleration and a source voltage set at 20 kV and 18.7 kV, respectively. For each spectrum, 240 laser shots in 40-shot steps were generated from different areas of the sample spot and analyzed using the default settings. The obtained protein profiles were analyzed by using a Flex Analysis and a Biotyper RTC 3 software. Therefore, the mass spectra, which were generated by MALDI-TOF MS, are multivariate data. Each mass signal is from a distinct molecular dimension. The multivariate statistical processes are applied to differentiate between bacterial strains. The principal component analysis (PCA) was used to reduce the dimensionality and keep the original information. The peaks of each MALDI-TOF MS spectrum were the basis of the PCA analysis. The peaks can be of proteins or peptides. The PCA led to the formation of assembled groups of spectra exhibiting similar characteristics and differences. A 2D or a 3D coordinate system can be generated with the data. However, the 2D system is more recommended since it plots PC1 against PC2 and offers in most cases, more than 80% of the total variance between the studied spectra. Besides, the hierarchical relationship between the isolates was investigated by establishing the dendrogram using the MALDI Biotyper Compass Explorer software that adopts default settings as per the manufacturer's instructions. The analysis by PCA and dendrogram was performed according to the standard operating procedure of the instrument and software. The spectra generated using triplicates were processed for smoothing and subtracting the baseline. Spectra with a strong background noise or high/low intensities were not used and then excluded. A main spectral projection (MSP) was created with the automated MSP creation functionality within the MALDI Biotyper 3.0 software by using the considered spectra. Indeed, the MSP provides information on the means of the peak frequencies, peak masses, and peak intensities. The generated MSPs for each isolate were fed to the functionality of PCA or dendrogram for analysis and generating graphs.

### 2.7 Biosurfactants production

5% (v/v) diesel was used as the carbon source to produce the biosurfactants in MSM liquid medium. The production medium consisted of 19 mL MSM liquid medium supplemented with 1 ml diesel in a 50 mL falcon tube, tightly sealed with PARAFILM® foil and incubated at 30°C, in a shaker set at 200 rpm for one week or two in dark, as specified with results. The cultures were inoculated with a suspension of cells from colonies of each isolate, formed overnight on LB plates. The initial cell density at the inoculation time corresponded to an optical density (OD) at 600 nm of 0.15. The cell density and the growth of each strain was evaluated by the determination of the colony-forming unit (CFU).

### 2.8 Colony-forming Unit (CFU) determination

CFU was determined by plating 100 µL of serial dilutions of the cultures on LB plates. The dilution corresponding to a number of colonies between 30 and 100 was considered. Then the CFUs were calculated for each ml of the corresponding culture.

### 2.9 Determination of the diesel solubilization activity

The procedure employed to determine the solubilization activity produced by each strain was based on the method described by Mnif et al. (2013) [24] using diesel. Biosurfactants solution was prepared by centrifugation of 1 mL of the cultured MSM (10,000 rpm for 5 min.). The produced supernatant was separated (0.9 mL) and added to 10 mL of Tris-HCl 20 mM (pH 7.0). Then, 0.2 mL of diesel was added to the tube to have a final 2% (v/v) diesel. Control Number 1 corresponded to a similar mixture except that 0.2 mL Tris-HCl buffer was used instead of diesel to determine the quantity of diesel solubilized by the strains in the culture medium. Control Number 2 was performed using 0.9 ml Tris-HCl instead of the culture's supernatant (without biosurfactants) and 0.2 mL diesel to determine the spontaneous solubilization of diesel in the aqueous phase. All tubes were incubated overnight at a vertical position in a shaker set at 30°C and 300 rpm, in the dark. After incubation, tubes were left out for 0.5 h to separate the diesel top layer. Then, 4 mL of the aqueous phase were mixed with an equal amount of pure hexane for extraction of diesel with vigorous vortexing during 2 min and then centrifugation at 4,500 rpm for 15 min. The optical density of the hexane phase was measured at 295 nm. Hexane was used as a blank. The concentration of diesel in the hexane phase was calculated from the slope of a calibration curve of different concentrations of diesel in hexane, ranging from 0.3 to 1.25 µL of diesel/mL. The amount of solubilized diesel and percent of solubilization were calculated using equations 1 and 2:

(1) Solubilizeddiesel=ODofthesupernatant−ODofcontrol1−−ODofcontrol2

The OD values were obtained from the calibration curve.

(2) %Solubilization=(Solubilizeddiesel/Initialdieselconcentration)×100

The initial concentration of diesel was 18.349 µL/mL. The solubilization activity of the freeze-dried biosurfactants was determined in 1 mg solubilized in 0.9 mL Tris-HCl and a similar method was employed.

### 2.10 Determination of the emulsification activity

MSM cultured broth of each strain was centrifuged (10,000 rpm for 15 min). 1 mL of the supernatant was supplemented with 0.15 mL diesel. It was vortexed for 2 min and left for 1 h to separate the diesel from the aqueous phase [25]. The aqueous phase was used to measure the optical density at 400 nm. Control Number 1 was performed with the fresh MSM instead of culture broth. Control Number 2 was performed with the culture broth and 0.15 distilled water instead of 0.15 ml Diesel. The emulsification activity (EA) was calculated as units of emulsification per milliliter (EU/mL), where each 0.01 absorbance is considered as one activity unit according to Patil and Chopade, (2001 a, b; 2003) [26] [27] [28], as equation 3:

Emulsification Activity (EU/mL) = (OD both – OD control 1 – OD control 2 / 0.01) × dilution factor (3)

The emulsification activity of the freeze-dried biosurfactants was determined in 1 mg solubilized in 1 mL Tris-HCl and a similar method was employed.

### 2.11 Extraction of biosurfactants from the cultures

Biosurfactants were extracted from each culture broth after centrifugation at 10,000 rpm for 15 min at 5°C. The employed method was that described by [29]. The pH of the supernatants was adjusted to 2.0 using 6.0 M HCl, and the solution was incubated for 24 h at 4°C. Then, CHCl_3_/CH_3_OH (2:1) was added to an equal volume, vigorously mixed, and incubated overnight at room temperature. After centrifugation for 15 min at 10,000 rpm, the pellet was suspended in Milli-Q water. The concentrate was neutralized to pH 7.0 with 1 M NaOH solution, then freeze-dried.

### 2.12 Analysis of the freeze-dried biosurfactants by Fourier transform infrared (FTIR)

The dried extracts of biosurfactants of each culture were analyzed by FTIR using an FTIR Perkin Elmer 400 FT-IR/FT-NIR spectrometer. The spectra were recorded in the range of 400–4000 cm^−1^. The method was described by Alkaabi et al. (2018) and Oualha et al. (2019).

## 3 Results and discussion

### 3.1 Isolation of hydrocarbon-degrading bacterial strains from weathered hydrocarbons-samples

Since the objective of this research was to establish a collection of bacterial strains isolated from highly polluted soils with weathered hydrocarbons in Qatar, we developed the strategy of isolation and screening shown in Fig. 1. It is expected that the diversity of the hydrocarbon-degrading bacterial strains is affected by the nature and composition of the carbon source. The weathering status of the hydrocarbons and their adsorption to the soil matrix should affect their availability, and thus the produced biosurfactants by the adapted bacterial community [30]. A total of 19 bacterial strains were isolated from the different locations (Table 1). All these strains are expected to be highly adapted to weathered hydrocarbons and tolerant to high toxicity exhibited by the 5% diesel employed in the enrichment and isolation steps.

### 3.2 Identification of the isolated strains by using the MALDI-TOF MS technique

**Fig. 2:**
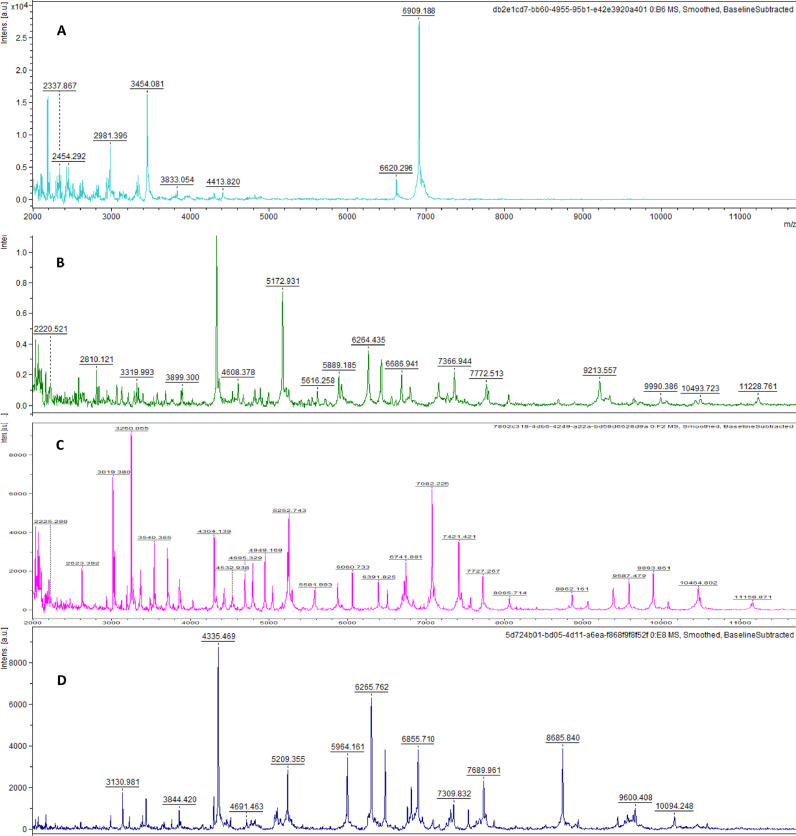
Examples of MALDI-TOF spectra as generated by the Bruker software for A) Bacillus subtilis (SA28), B) Bacillus cereus (SA17), C) Bacillus licheniformis (S33) and D) Lysinibacillus boronitolerans (SA3).

The 19 isolated strains were identified by MALDI-TOF MS protein profiling, using the available database in the used apparatus. Indeed, a MALDI score and a reproducible protein profile were obtained for each strain by MALDI TOF MS profiling (Table 2). Fig. 2 shows an example of protein profiles obtained with different strains. Proteins with an *m/z* in the range between 2000 and 20 000 m/z are used to identify bacterial strains based on individual mass peaks corresponding to specific ribosomal proteins of distinct types of microorganisms, compared to a database available in the software. It is interesting to notice that 15 isolates were identified at the level of *Bacillus* genus (five *Bacillus subtilis*, four *Bacillus cereus*, two *Bacillus atrophaeus*, one *Bacillus licheniformis*, two *Bacillus sonorensis,* and one *Bacillus mojavensis*). Three isolates belong to the *Lysinibacillus* genus (two *Lysinibacillus boronitolerans*, and one *Lysinibacillus fusiformis*). One isolate was *Enterococcus faecium*.

**Table 2:** Identification of the 19 isolated strains based on their MALDI scores

Strain code	MALDI Score	Identification	Location of isolation
S5	1.88	*Bacillus subtilis*	*Dukhan Sealine*
S24	1.96	*Bacillus cereus*	*AWS*
S27	2.08	*Bacillus subtilis*	*AWS*
S32	2.00	*Bacillus cereus*	*Dukhan DS2*
S33	2.07	*Bacillus licheniformis*	*Dukhan DS2*
SA2	1.8	*Bacillus atrophaeus*	*Dukhan Sealine*
SA3	2.07	*Lysinibacillus boronitolerans*	*Dukhan Sealine*
SA4	2.06	*Lysinibacillus fusiformis*	*Dukhan Sealine*
SA6	2.14	*Bacillus subtilis*	*Dukhan DS1*
SA9	1.76	*Bacillus sonorensis*	*Dukhan IZ*
SA10	1.84	*Lysinibacillus boronitolerans*	*Dukhan IZ*
SA11	1.81	*Bacillus sonorensis*	*Dukhan IZ*
SA12	2.42	*Enterococcus faecium*	*Dukhan IZ*
SA14	1.84	*Bacillus atrophaeus*	*Dukhan IZ*
SA16	1.92	*Bacillus subtilis*	*Dukhan IZ*
SA17	2.19	*Bacillus cereus*	*Dukhan DS1*
SA28	1.95	*Bacillus subtilis*	*Dukhan DS2*
SA29	1.8	*Bacillus mojavensis*	*Dukhan DS2*
SA31	2.06	*Bacillus cereus*	*Dukhan DS2*

In literature, some strains of *Bacillus cereus* were described as hydrocarbon-degrading bacteria [31, 18]. Other studies reported the biosurfactants of *B. cereus* [32]. Biosurfactants of *Bacillus lichenifomis* (*B. licheniformis*) JF-2 were formulated for the petroleum industry [33]. *Bacillus mojavensis* is known for forming biofilms and producing biosurfactants [34]. Surfactants produced by *Bacillus atrophaeus* were reported by Rodriguez et al., (2018) [35]. Bacillus sonorensis is known as a hydrocarbon-degrading bacterium *[**31**]* and biosurfactant producer *[**36**]*. The genus *Lysinibacillus* is distinguished but relatively close to *Bacillus* as phylogeny, composition of peptidoglycan, and physiology [37]. *Lysinibacillus fusiformis* and *Lysinibacillus boronitolerans* were originally known as *Bacillus fusiformis* and *Bacillus boronitolerans* before 2007 [37]. Lin et al., (2010) [38] reported the potential of *Bacillus fusiformis* in the biodegradation of naphthalene. Recently, Li et al. (2020) [39] showed the survival of *Lysinibacillus fusiformis* in petroleum environments. While *Lysinibacillus sphaericus* and *Geobacillus sp* are also shown activity in the biodegradation of petroleum hydrocarbons and biosurfactants production [40], however, the involvement of *Lysinibacillus* (*Bacillus*) *boronitolerans* in oil hydrocarbons degradation was never reported. It was only associated with tolerance to boron [37]. Ozyurek and Bilkay (2017) [41] reported the isolation of *Enterococcus faecium* as a hydrocarbon-biodegrading bacterium in crude oil, waste mud pit, and drilling fluid.

These results also show that some well-known bacteria with their high degradation activity of oil hydrocarbons were not isolated from the highly weathered oil-soils used in this work. The bacterium *Pseudomonas aeruginosa* was not found within this new collection of hydrocarbon-degrading bacterial strains. By considering the potential of biosurfactants to be applied to oil degradation and recovery, Rhamnolipids produced by Alcaligenes eutrophus have been shown to be effective in increasing the solubility of polychlorinated biphenyls (PCBs) and their mineralization [42]. Commercialized Rhamnolipids of Pseudomonas aeruginosa (P. aeruginosa) enhanced extraction of hexadecane residues in a sand column, compared to sodium dodecyl sulfate (SDS) and sorbitan monooleate, both are synthetic surfactants [43]. Rhodococcus ST-5 and Badus AB-2 surfactants allowed recovery of 95% of the crude oil residues. Biosurfactants of Bacillus subtilis were reported but not in the field of oil remediation or recovery [44]. B. licheniformis is also able to produce biosurfactants. Crude biosurfactant production by B. licheniformis can reach 1 g/L, with emulsification power increased up to 96% [45]. Bacillus flexus was also able to produce biosurfactants, even with a low emulsification index.

### 3.3 Differentiation of the isolated strains using PCA and dendrogram analysis

**Fig. 3:**
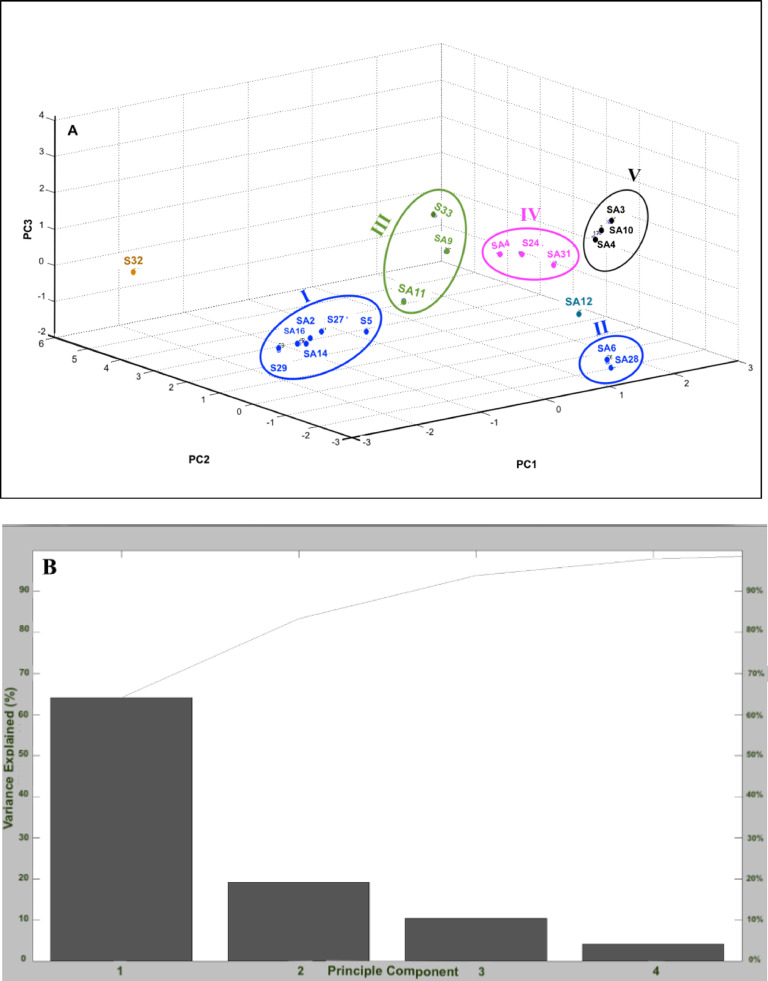
Classification of the studied strains using PCA(A) PCA plot and (B) percentage of variance explained.

Further information on the relationships between the closely related isolates can be obtained upon combining the MALDI-TOF MS analysis and PCA. By reducing the dimensions of objects being studied, linear combinations could be created for variables, representing the studied objects. The PCA results are shown in Fig. 3A. The PCA clustering revealed large biodiversity between the studied strains at the protein level. The total variance of the 10 principal components is shown in (Fig. 3B). PC1 (34%), PC2 (25.5%) and PC3 (10.5%) combine to show a total of 70% variability in the data. Using the first three principal components, five clusters were obtained. The distances between the clusters indicate the variations at a group level, while the distance between the strains (within each cluster) shows the differences in protein profiles at the strain level. Clusters I and II include *B. subtilis* strains (S5, S27, SA16, SA6, and SA28). The strain *B. mojavensis* SA29 falls within-cluster I, indicating high similarity in their protein profiles. Similarly, *B. licheniformis* (S33) falls within Cluster III that includes the two *B. sonorensis* (SA9 and SA11). Whereas, cluster IV includes three *B. cereus* (SA17, S24, and SA31). Cluster V includes the three *Lysinibacillus* strains (SA3, SA4, and SA10). Interestingly, the strain *B. cereus* S32 is located at a huge distance from Cluster IV, demonstrating a large variation in its protein profile in comparison to the other *B. cereus* strains in cluster IV. Similarly, the *Enterococcus faecium* SA12 is distinct from any of the five clusters.

**Fig. 4:**
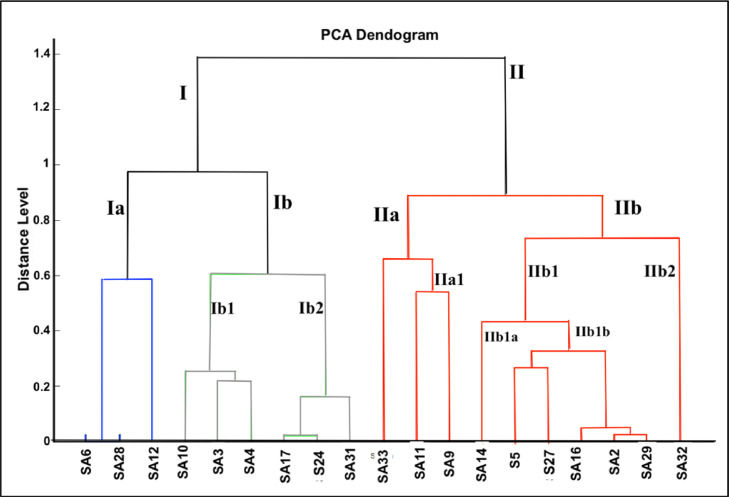
PCA dendrogram of the studied strains.

The hierarchical relationship between the isolates was investigated by establishing the class dendrogram (Fig. 4). The dendrogram revealed two major clusters (I, II). Cluster I is formed of 9 strains and cluster II of 10 strains. In each cluster, two distinct clades are distinguished. Cluster I includes clades Ia formed with two strains of *Bacillus subtilis* (SA6 and SA28) and the strains *Enterococcus faecium* (SA12). Clade Ib is further subdivided into the sub-clades Ib1 formed with the three *Lysinibacillus* strains (SA3, SA4, and SA10), and sub-clade Ib2 formed with the three *Bacillus cereus strains* (SA17, S24, SA31). Cluster II includes 2 clades (IIa, IIb). Clade IIa is formed with the strain *Bacillus licheniformis (*S33) and clade IIa1 is formed with the two *Bacillus sonorensis* strains (SA9 and SA11)*.* Clade IIb2 includes one *Bacillus cereus* strain (SA32). Clade IIb1 is further divided into sub-clade IIb1a formed with the two *bacillus subtilis* strains (S5 and S27) and sub-clade IIb1b formed with the strains *Bacillus subtilis (*SA16), *Bacillus mojavensis* (SA2), and *Bacillus atrophaeus* (SA29). The phyloproteomic classification of the studied strains allowed the differentiation and separation of strains belonging to the same *Bacillus* species*.* Two *B. subtilis* strains (SA6 and SA28) were classified in clade Ia while other strains (SA5, S27, and SA16) were classified in IIbIa. Similarly, three strains belonging to *Bacillus cereus* (SA17, S24, and SA31) belong to cluster Ia, while the strain B*acillus cereus* SA32 is under cluster IIb. The approach of using protein profiles may be informative in differentiating strains belonging to the same species [46]. Indeed, MALDI-TOF MS could be used to characterize isolates with much higher precision than that of 16S rRNA sequencing. The resolving capability of MALDI-TOF MS is higher than that of 16S rRNA sequencing because it covers a wider range of proteins than the 16S ribosomal subunit. The ability to accurately characterize differences on the strain level is valuable in understanding differences among redundantly isolated strains identified as the same species, while isolated at different occurrences, such as those isolated from different contaminated soils. MALDI-TOF MS is useful for the identification and grouping of isolates based on the strain-level variations [47]. Here, it allowed differentiation and categorization of newly isolated strains from highly weathered oily soils. It also demonstrates the high diversity of the strains as a consequence of their adaptation to harsh conditions (chemical and physical).

### 3.4 Diesel solubilization and emulsification activities of the isolated strains

All the isolated strains were cultured in the MSM medium containing 5% (v/v) diesel as the sole carbon source. After one week and 2 weeks of incubation at 30°C, the supernatants were collected and used to assess the diesel solubilization (SA) and emulsification (EA) activities of each strain (Table 3). These results show that SA and EA activities are fluctuating in a wide range of 1.4 U/ml to 42.1 U/ml for EA and 1.17 (%) to 8.6 (%) for SA. Moreover, some strains exhibited high EA activity and low SA or vice versa. They confirm the diversity of the strains. The growth of the strains was also varying from 11 to 99 10^8^ CFU/mL, which was almost the same or strongly reduced after 2 weeks of incubation. The specific activity for EA and SA activity would be the characteristics of each strain. The strains *Bacillus subtilis* S5 exhibited the highest growth (99 10^8^ CFU/mL) and SA (8.6%) and the second-highest EA (31.7 U/mL). The strain *Lysinibacillus fusiformis* SA4 exhibited the highest EA (42.1 U/mL) but low SA activity (1.17%) and low growth (11 10^8^ CFU/ml). However, the strain *Lysinibacillus boronitolerance* SA10 produced 2.6 (%) as SA and 23.2 U/mL as EA with 33 10^8^ CFU/mL. In contrast, *Lysinibacillus boronitolerance* SA3 providing a similar growth (30 10^8^ CFU/mL) produced 6.7 U/mL of EA and 4.6 % as SA. All these results confirm the high diversity among the collection of the isolated strains from weathered oily-soils. The diversity of the biological activities of the strains, as growth and diesel solubilization and emulsification does not reflect their ordering shown in the dendrogram of Fig. 3. Indeed, each of these activities can modify the succession of the strains differently. Besides the diversity based on the protein profiles, a diversity based on the function can be established.

**Table 3:** Growth, diesel solubilization, and emulsification capacity, and specific activities of the isolated strain.

1 week incubation						2 weeks incubation				
Strains	CFU (10^8^/mL)	EA (U/mL)	SA (%)	EA/ CFU 10^−3^	SA/ CFU 10^−3^	CFU (10^8^/mL)	EA (U/mL)	SA (%)	EA/ CFU 10^−3^	SA/ CFU 10^−3^
S5	99 ± 5	32 ± 2	8.6 ± 0.4	341 ± 17	93 ± 5	37± 2	14.4 ± 0.7	4.2 ± 0.2	389 ± 19	114 ± 6
S24	75 ± 4	19 ± 1	2.4 ± 0.1	251 ± 13	32 ± 2	50 ± 3	6.7 ± 0.3	3.0 ± 0.2	134 ± 7	61 ± 3
S27	29 ± 1	28 ± 1	1.9 ± 0.1	966 ± 48	66 ± 3	30 ± 2	15.8 ± 0.8	1.4 ± 0.1	527 ± 26	48 ± 2
S32	21 ± 1	11 ± 6	1.97 ± 0.1	533 ± 27	94 ± 5	7 ± 1	2.9 ± 0.2	1.6 ± 0.1	414 ± 21	230 ± 12
S33	23 ± 1	17 ± 1	2.6 ± 0.1	757 ± 38	114 ± 6	27 ± 1	1.3 ± 0.1	2.4 ± 0.1	48 ± 2	90 ± 5
SA2	18 ± 1	1.4 ± 0.1	4.3 ± 0.2	78 ± 4	238 ± 12	15 ± 1	6.8 ± 0.3	5.7 ± 0.3	453 ± 23	378 ± 19
SA3	30 ± 2	6.7 ± 0.3	4.86 ± 0.2	223 ± 11	162 ± 8	37 ± 2	18.2 ± 0.9	2.6 ± 0.1	492 ± 25	71 ± 4
SA4	11 ± 1	42 ± 2	1.2 ± 0.1	3827 ± 191	106 ± 5	1.2 ± 0.1	2.3 ± 0.1	3.0 ± 0.2	2300 ± 115	3040 ± 152
SA6	75 ± 4	22 ± 1	4.5 ± 0.2	217 ± 11	127 ± 6	40 ± 2	28 ± 1	9.5 ± 0.5	693 ± 35	87 ± 4
SA9	17 ± 1	10 ± 1	5.8 ± 0.3	565 ± 28	225 ± 11	10 ± 1	12.4 ± 0.6	2.4 ± 0.1	1240 ± 62	242 ± 12
SA10	33 ± 2	23 ± 1	2.6 ± 0.1	703 ± 35	77 ± 4	27 ± 1	4.3 ± 0.2	1.3 ± 0.1	159 ± 8	48 ± 2
SA11	75 ± 4	9.5 ± 0.5	6.0 ± 0.3	87 ± 4	81 ± 4	33 ± 2	19 ± 1	2.2 ± 0.11	576 ± 29	68 ± 3
SA12	38 ± 2	26 ± 1	1.3 ± 0.1	687 ± 34	34 ± 2	42 ± 2	19 ± 1	3.4 ± 0.2	457 ± 23	81 ± 4
SA14	38 ± 2	3.2 ± 0.2	5.7 ± 0.3	84 ± 4	149 ± 7	36 ± 2	10.3 ± 0.5	2.5 ± 0.1	286 ± 14	68 ± 3
SA16	22 ± 1	34 ± 2	3.1 ± 0.2	1550 ± 78	142 ± 7	20 ± 1	5.8 ± 0.3	3.5 ± 0.2	290 ± 15	174 ± 9
SA17	33 ± 2	8 ± 0.4	5.1 ± 0.3	248 ± 12	153 ± 8	50 ± 3	27 ± 1	7.3 ± 0.4	548 ± 27	67 ± 3
SA28	26 ±1	21 ± 1	7.5 ± 0.4	823 ± 41	287 ± 14	20 ± 1	18 ± 1	3.2 ± 0.2	900 ± 45	162 ± 8
SA29	25 ± 1	17 ± 1	4.0 ± 0.2	668 ± 33	161 ± 8	60 ± 3	20. ± 1	2.2 ± 0.1	337 ± 17	36 ± 2
SA31	32 ±1.6	3.8 ± 0.2	4.8 ± 0.2	119 ± 6	149 ± 7	18 ± 1	23 ± 1	2.5 ± 0.1	1272 ± 64	137 ± 7

Bacteria employ biosurfactants as one of multiple adaptation mechanisms to use hydrocarbons as substrates. The adaptations largely express specific physiological responses to specific microenvironments of the cell and its nutritional requirements [48]. Indeed, some bacteria developed a strategy of pseudo-solubilization to increase the solubility of poorly soluble hydrocarbons. Therefore, they produce a high capability of self-assembly in micelles, hemi-micelles or aggregates, by using highly dynamic low-molecular-mass molecules of biosurfactants. However, other bacteria develop a direct interaction with hydrocarbons by a different tool through the wall-bound biosurfactants. Thus, the cell surface becomes appropriately hydrophobic. Indeed, the high molecular mass molecules are called bioemulsifiers, which adsorb tightly to the hydrocarbons and thus increase their apparent solubility by covering them in the aqueous phase. Biosurfactants share few traits, although their wide variety of specialization and mechanisms to deal with hydrocarbons. All mechanisms are around the interactions between the three phases (cell physiology, cell surface, hydrocarbons that are the substrates for the cell). To achieve the goal of passing the hydrocarbons across the wall, the cell develops reversible and temporary modifications of the membrane adapted to the nature, composition, and type of hydrophobicity of the available substrates, in addition to making the hydrocarbons more soluble [48]. It is not excluded that a “substrate effect” can be developed during the growth of the cell. However, the synthesis pathways of most of the biosurfactants are not yet elucidated. In contrast, the hydrophobic substrates are known to influence the structural variations of biosurfactants to make them particularly active on the same substrate. In addition, it is now established that biosurfactants stimulate the growth of their producing strains, playing a vital role in the interaction between the microbial communities and their micro-environment [48].

### 3.5 Production and stability of the biosurfactants activity during growth of the strains

The concomitant production of the EA and SA by four selected strains based on the results of Table 3 was investigated. The strain *Bacillus subtilis* S5 is characterized by high growth and SA and EA activities. The strain *Bacillus subtilis* SA6 exhibited lower growth and activities than *Bacillus subtilis* S5. The strain SA28 is *Bacillus subtilis* strain characterized by a much higher specific activity of SA and EA activities. The strain SA9 is *Bacillus sonorensis* selected because it is characterized by shining colonies, embedded in the extensive production of exopolymeric substances.

**Fig. 5:**
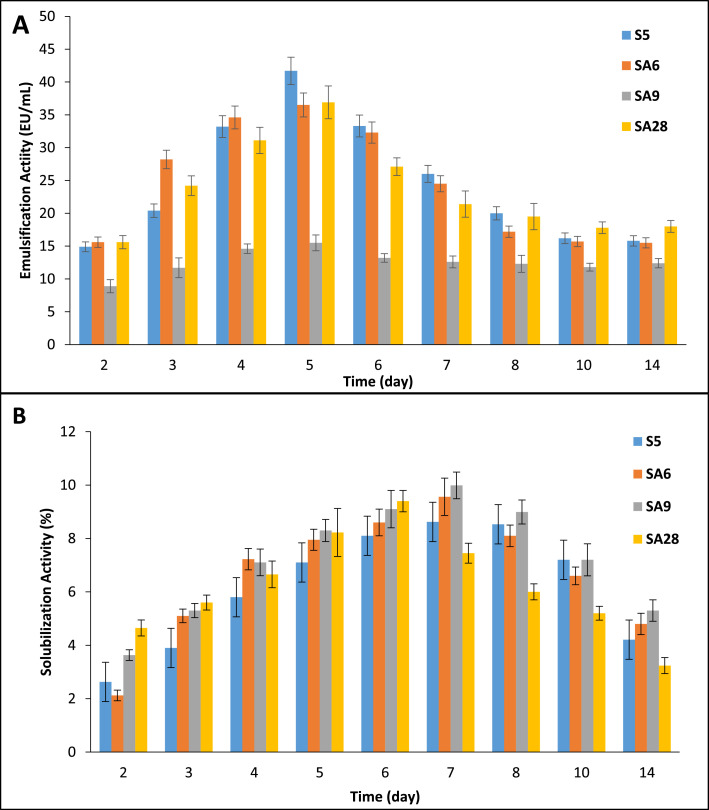
Concomitant production of A) EA and B) SA activities by the 4 selected strains.

The SA and EA activities of the four strains were evaluated during dynamic growth in 5% diesel-MSM. Results are shown in Figure 5.

The four strains exhibited, during their growth, a maximum of production of the emulsification activity after 5 days incubation, while their solubilization activities were maximal after 7 days incubation. The activities were remarkably unstable with a continuous and rapid decrease after reaching the maximum. The behavior of the 4 *Bacillus* strains was almost similar.

This result confirms that all the bacterial strains develop reversible and temporary modifications of the membrane adapted to the available substrates while making a more continuous activity of solubilization of the hydrocarbons. The concomitant production of both activities is necessary to achieve the goal of passing the hydrocarbons across the wall. The bio-emulsification touching the cell surface structure and functionality is normal in that it attains its highest activity during the vegetative growth of the cells, after which, the cells enter into sporulation phase with loss of and disintegration of the cell membranes, and thus of the related activities. The solubilization activity can continue to be produced by the sporulating cells or the remaining vegetative ones.

### 3.6 Analysis of the freeze-dried biosurfactants by Fourier transform infrared (FTIR)

**Fig. 6:**
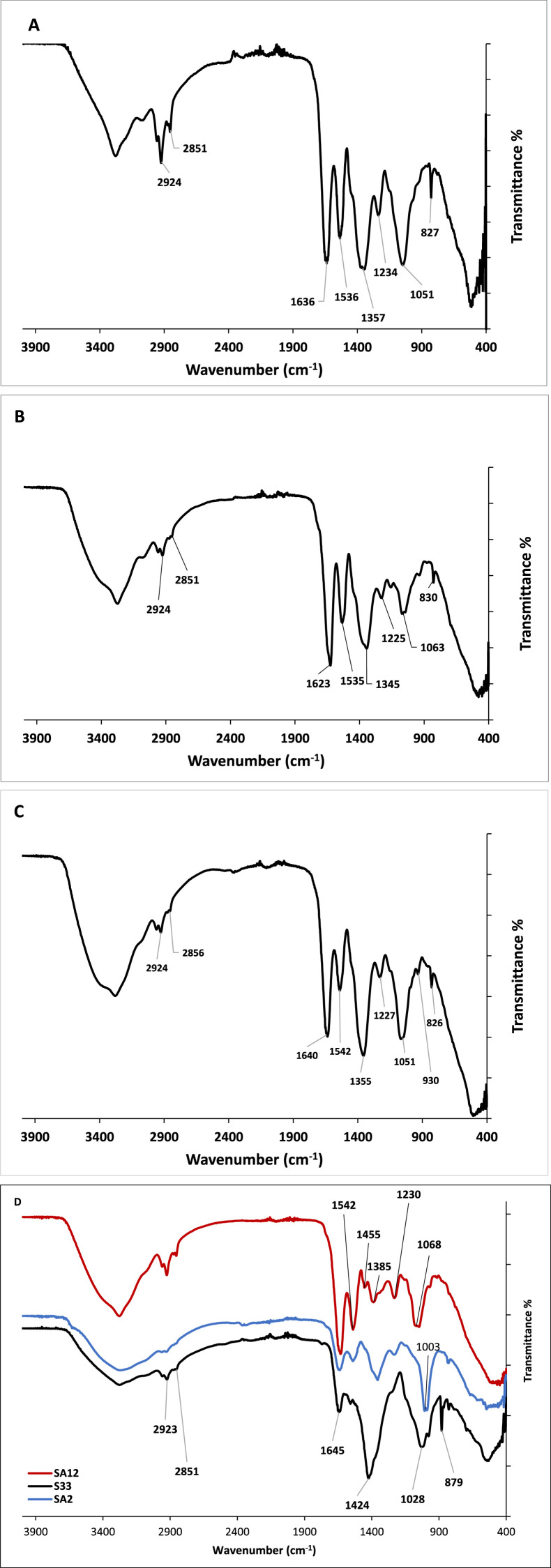
Representative FTIR spectra of biosurfactants of: A) Group 1, B) Group 2, C) Group 3, D) Bacillus licheniformis (S33), Bacillus atrophaeus (SA2) and Enterococcus faecium (SA12).

The FTIR absorption spectra of the biosurfactants produced by the studied strains revealed the presence of protein, polysaccharide, ester, and carbonyl groups indicating the presence of lipopeptides (Fig. 6 A-D). The strong absorption peak at 3270 cm^−1^ corresponds to the stretching vibrations of –NH and –OH groups related to peptides [49]. The absorption peaks in the range from 2960 cm^−1^ and 2860 cm^−1^ are due to the asymmetric and symmetric stretching of the methylene groups of lipids (–CH_2_) [50, 51]. The absorption peak at 1640 cm^−1^ - 1630 cm^−1^ can be attributed to the CO-NH bend (due to the stretching vibrations of C=O and C-N groups), which confirms the presence of a peptide group in the biosurfactants [52]. The absorption peaks at 1451 cm^−1^ and 1360 cm^−1^ appear due to the presence of alkyl (-CH_2_ and –CH_3_) groups [53]. The presence of ether moiety is confirmed due to the presence of an absorption peak at 1230 cm^−1^. Hence, the biosurfactants were expected to contain fatty acids and peptide moieties indicating their lipopeptides nature [49].

### 3.7 PCA analysis of the FTIR spectra

**Fig. 7:**
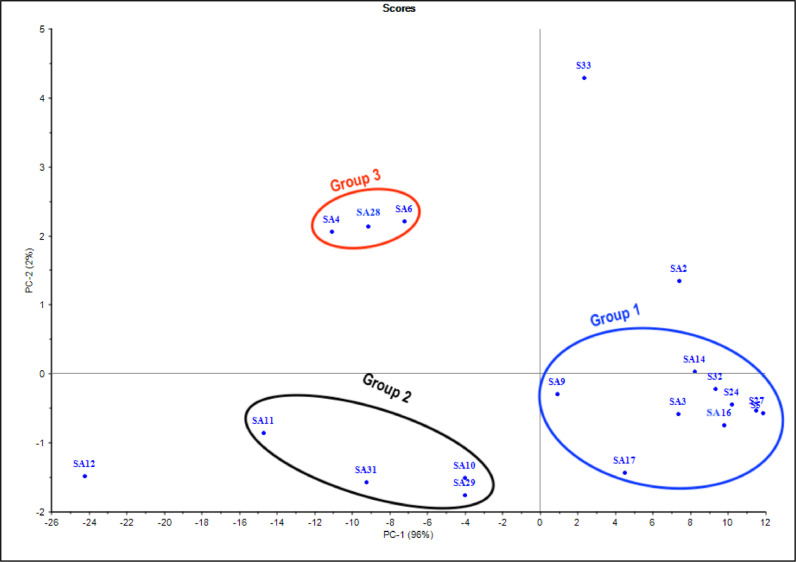
PCA classification for the FTIR spectra of the biosurfactants obtained from the studied strains.

To have more insights into the variations in the obtained FTIR spectra for the biosurfactants, PCA analysis was performed (Fig. 7). The FTIR-PCA clustering revealed three main groups. Group 1, the largest one, contained the biosurfactants obtained from nine bacterial strains; three *B. subtilis* (S5, S27 and SA16), three *B. cereus* (SA17, S24 & S33), one *B. sonorensis* (SA9)*,* one *B. atrophaeus* (SA14), and one *L. boronitolerans* (SA3). Group 2 contained biosurfactants of one *B. cereus* (SA31), one *B. sonorensis* (SA11), one *B. mojavensis* (SA29), and one *L. boronitolerans* (SA10). Group 3 contained biosurfactants of two *B. subtilis* (SA6 & SA28) and one *L. fusiformis* (SA4). Each of the biosurfactants of *Enterococcus faecium* (SA12), *B. atrophaeus* (SA2) and *B. licheniformis* (S33) is located in separate large distances from any of the groups.

The FTIR bands at 2924 cm^−1^, 2850 cm^−1^ were preserved in all FTIR spectra of the studied, biosurfactants. However, variations were observed in FTIR bands in the region from 800 cm^−1^ to 1640 cm^−1^. Obvious shifts in the amide I peaks from 1645 cm^−1^ to 1623 cm^−1^ were observed in the FTIR spectra of the biosurfactants assembled in PCA-Group 2 (Fig. 7 & Fig. 6B). These shifts are attributed to changes in protein secondary structure [54, 55]. Moreover, the peaks at 1154 cm^−1^ that may be ascribed presence of ester bonds [56] are characteristics of the biosurfactants categorized in PCA-Group 2 (Fig. 7 & Fig. 6B). The weak bands at 930 cm^−1^ which corresponds to phosphorus and oxygen stretching in aliphatic and aromatic molecules [57] were clearly observed in the FTIR spectra of biosurfactants clustered in PCA-Group 3 containing biosurfactant produced by *B. subtilis* (SA6 & SA28) and *L. fusiformis* (SA4) (Fig. 6C), which may indicate the presence of phosphate in these biosurfactants. The FTIR peak at 1028 cm^−1^ attributed asymmetric and symmetric C–O–C stretching of ester [58, 59] was only observed in the FTIR spectra of the biosurfactants of B. lichenifomis S33. The FTIR peak 1003 cm^−1^ observed only in the FTIR spectra of *B. atrophaeus* SA2 (Fig. 6D) may be assigned to O–C–O extend vibrations of carboxylic acids. This is a remarkable indication of the oxidation of the hydroxyl groups in the hydrolysates from the medium peptides [60].

Indeed, even with the same group of isolates clustered based on their protein profiles, the corresponding biosurfactants are clustered in different groups based on their FTIR spectra. This means that each isolate was able to adapt differently its biosurfactant composition in response to the existing weathered oil components and the weather conditions. However, the three strains, *Enterococcus faecium* (SA12), *Bacillus mojavensis* (SA2) and *B. licheniformis* (S33) which were not within any of the isolates’ clusters produce biosurfactants with structure that are strongly different from those of all the clustered ones, although several similarities were observed. The strain S32, which is not clustered with the other isolates produces biosurfactants highly similar to those of FTIR-cluster I, grouping 9 out of the 19 surfactants.

## 4 Conclusion

The ability of the indigenous Qatari strains to produce biosurfactants with great potential to enhance the biodegradation of weathered hydrocarbons was investigated. The obtained findings showed that two types of adaptations occur with hydrocarbons degrading bacteria in the weathered-oily soils, one related to the bacterial cell composition maintaining the biosurfactants composition and one to the biosurfactants which is the primary tool employed by the bacterial cell to interact with the weathered oil. Indeed, the phyloproteomic classification of the studied strains allowed the differentiation and separation of strains belonging to the same *Bacillus* species. High diversity of emulsification and solubilization activities were recorded among biosurfactants produced by the studied strains. Moreover, combining FTIR data with PCA analysis resulted in further classification of the biosurfactants produced by the studied bacterial isolates.

## Declaration of Competing Interest

The authors declare that they have no known competing financial interests or personal relationships that could have appeared to influence the work reported in this paper.
